# Medical News and Miscellany

**Published:** 1898-11

**Authors:** 


### Medical News and Miscellany.

---

**Dr. R. W. Hix** has removed from Effie to Vernon, Texas.

---

**Died,** at Yoakum Texas, August 18, 1898, Dr. D. W. Boyd, a prominent physician of that city.

---

**For Sale or Exchange.**—One set Allen's Anatomy, "portfolio"; one Howard D. B. Speculum, nickel plated. Address, P. O. Box 158, Austin, Texas.

---

**Wanted**—Agents for "History of the Spanish-American War," by Henry Watterson. A complete, authentic history; illustrated with over 76 full-page half-tones and many richly colored pictures. Large royal octavo volume, superb outfit, postpaid for only 50 cents (stamps taken). Most liberal terms given. The greatest opportunity of the year. Address: THE WERNER COMPANY, Akron, Ohio.

---

**The Southwestern Medical Record,** Houston, says that Surgeon General Sternberg has selected two medical men from Houston for work in Cuba and Poto Rico—neither of whom is "elegible for membership in any of our regular County or State Medical Societies." "One has been out of practice many years and is a great advocate of the doso metric system of therapeutics, while the other is unclassified." Give us their names Brother Red.

SHERMAN, TEXAS, Oct. 24, 1898.

DEAR DOCTOR:—The North Texas Medical Association will meet in Paris, Texas, December the 13th, 1898, and continue in session three days. The program and arrangements give promise of an interesting and profitable session of this the largest and best working Association in the State. Come and enjoy the meeting with us.

Very truly yours,

R. D. POTTS, President, Bonham.

R. F. MILLER, Secretary.

---

**Dr. F. Hoyt Pilcher,** Superintendent asylum for idiotic and imbecile youth at Winfield, Kansas, who, it will be remembered, emasculated some ten of the weak minded boys for confirmed habit of onanism, and greatly improved them mentally and physically, and for doing which some of the "unco gude" tried to have him indicated, sends us his ninth Biennial Report. From it we see that he has since operated on forty-seven additional unfortunates and with good results. The doctor is a benefactor; he is a pioneer in the work.

---

**At the Twenty-Fourth Annual Meeting** of the Mississippi Valley Medical Association held at Nashville, Tenn. Oct. 11–16, 1898, the following officers were elected for the ensuing year:

President, Dr. Duncan Eve, Nashville, Tenn.; First Vice-President, Dr. A. J. Ochsner, Chicago, Ill.; Second Vice-President, Dr. J. Morfit, St. Louis, Mo.; Secretary, Dr. Henry E. Tuley, Louisville, Ky. (111 W. Ky. St.); Treasurer; Dr. Dudley S. Reynolds, Louisville, Ky. Next place of meeting, Chicago.

Chairman of Committee on Arrangements: Dr. Harold N. Moyer.

Time of meeting, October, 1899, date to be determined by the executive officers and Chairman of the Committee on Arrangements.

---

**The Texas Clinic,** a journal devoted to clinical medicine and surgery, J. B. Shelmire, M. D., editor, Bradfield Bros. publishers, Dallas, Texas. Subscription $1 a year.

We have received Vol. 1, No. 1 of the *Texas Clinic*, a neat little publication of fifty-six pages, just established at Dallas. Dallas is a flourishing city of some 50,000 inhabitants, and surely ought to be represented in the medical press. Dallas has a most excellent body of professional men and we are pleased to see that many of the well-known practitioners of the city are enlisted as collaborators in the interest of *The Texas Clinic*.

We send greeting and wish *The Texas Clinic* success.

